# Assessing the Genetic Background and Selection Signatures of Huaxi Cattle Using High-Density SNP Array

**DOI:** 10.3390/ani11123469

**Published:** 2021-12-06

**Authors:** Jun Ma, Xue Gao, Junya Li, Huijiang Gao, Zezhao Wang, Lupei Zhang, Lingyang Xu, Han Gao, Hongwei Li, Yahui Wang, Bo Zhu, Wentao Cai, Congyong Wang, Yan Chen

**Affiliations:** 1 Institute of Animal Science, Chinese Academy of Agricultural Sciences, Beijing 100193, China; junma96@163.com (J.M.); gaoxue76@126.com (X.G.); lijunya@caas.cn (J.L.); gaohuijiang@caas.cn (H.G.); wangzezhao1@163.com (Z.W.); zhanglupei@caas.cn (L.Z.); xulingyang@caas.cn (L.X.); gaohan0412@126.com (H.G.); lihongweicaas@163.com (H.L.); wang1434243198@163.com (Y.W.); zhubo@caas.cn (B.Z.); wtaocai@gmail.com (W.C.); 2 Beijing Lianyu Beef Cattle Breeding Technology Limited Company, Beijing 100193, China; cjiangling@163.com

**Keywords:** Huaxi cattle, genetic diversity, population structure, genetic relationships, selection signature

## Abstract

**Simple Summary:**

Evaluating population genetic background and genomic selection signatures can provide important insights into the uniqueness and the genetic relationship among breeds. In this study, we analyzed the genetic diversity, population structure, and phylogenetic relationships between Huaxi cattle and its ancestors. The results showed that Huaxi cattle has formed its own unique genetic structure features, which could be clearly distinguished from its ancestors and similar populations. Moreover, some genes associated with important economic traits, including growth and development, reproduction, milk quality, and immune response, were identified by scanning potentially selected genomic regions, which may play an essential role in the excellent environmental adaptability and growth performance of Huaxi cattle. Our study will contribute to the genetic evaluation and rational breeding strategy, and give an extensive reference for understanding the genetic basis of economically important traits in Huaxi cattle.

**Abstract:**

Huaxi cattle, a specialized beef cattle breed in China, has the characteristics of fast growth, high slaughter rate, and net meat rate, good reproductive performance, strong stress resistance, and wide adaptability. In this study, we evaluated the genetic diversity, population structure, and genetic relationships of Huaxi cattle and its ancestor populations at the genome-wide level, as well as detecting the selection signatures of Huaxi cattle. Principal component analysis (PCA) and phylogenetic analysis revealed that Huaxi cattle were obviously separated from other cattle populations. The admixture analysis showed that Huaxi cattle has distinct genetic structures among all populations at K = 4. It can be concluded that Huaxi cattle has formed its own unique genetic features. Using integrated haplotype score (iHS) and composite likelihood ratio (CLR) methods, we identified 143 and 199 potentially selected genes in Huaxi cattle, respectively, among which nine selected genes (*KCNK1*, *PDLIM5*, *CPXM2*, *CAPN14*, *MIR2285D*, *MYOF*, *PKDCC*, *FOXN3*, and *EHD3*) related to ion binding, muscle growth and differentiation, and immunity were detected by both methods. Our study sheds light on the unique genetic feature and phylogenetic relationship of Huaxi cattle, provides a basis for the genetic mechanism analysis of important economic traits, and guides further intensive breeding improvement of Huaxi cattle.

## 1. Introduction

Cattle are vital agricultural economy species worldwide, providing meat, milk, and labor for humans. China has abundant cattle genetic resources, with rough feeding tolerance, excellent meat quality, and strong adaptability [1]. For a long time, Chinese native cattle have been used as draft animals, ignoring the utilization of meat performance, and resulting in lower beef production performance compared to commercial cattle breeds. Hybridization is an important method for improving the desirable performance and overall genetic variation of cattle breeds [2]. Crossbreeding breeds combine the beneficial traits/genes of their purebred parental breeds, and have more superior production capacity than their parental inbred lines under the effect of heterosis [3,4]. Therefore, the introduction of excellent beef cattle breeds to cultivate specialized beef cattle breeds has become an important way to elevate the individual production efficiency of beef cattle.

**Figure 1:**
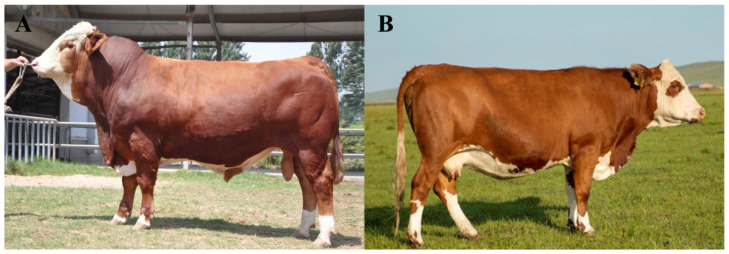
Photographs of Huaxi cattle. (**A**) bull and (**B**) cow.

Huaxi cattle, formerly known as “China Simmental beef cattle”, is a new specialized beef cattle breed in China, which consists of 24/32 Northern America beef Simmental cattle, 5/32 of dual-purpose Simmental cattle from Germany and Austria, 1/32 Charolais cattle, 1/32 Sanhe cattle, and 1/32 Mongolian cattle (Figure 1). The breeding of Huaxi cattle started in 1978, since then, they have displayed good performance in growth rate, feed conversion rate, slaughter rate, net meat rate, reproduction, resistibility, adaptability, and economic efficiency after 43 years of cross-breeding and continuous breeding. During the breeding process of Huaxi cattle, studies were carried out on its diverse important phenotypic traits. By fitting the growth curve with the body weight of 808 individuals at 0, 6, 12, and 18 months for a genome-wide association study (GWAS), several candidate genes, namely, *MYH10*, *RLF*, *ARHGAP31*, *SQOR*, and *TBCB*, were identified as associated with the growth and development traits [5]. Given the remarkable growth and weight gain of Huaxi cattle, Zhuang et al. found that the *RLF* gene had a significant effect on the average daily gain from birth to yearling by GWAS of 3996 Huaxi cattle [6]. Carcass traits are direct indicators reflecting the meat production ability of livestock and poultry. According to the slaughter measurement, the slaughter rate and net meat rate of 135 fourth-generation Huaxi bulls aged 20 to 24 months were 62.39 ± 1.67% and 53.95 ± 1.46%, respectively (unpublished data). In addition, Song et al. revealed the *PLAG1* gene as the candidate gene for knuckle, biceps, and shank [7], and Chang et al. identified that eight and seven SNPs were significantly related to carcass weight and bone weight, respectively [8]. Moreover, as a new specialized beef cattle breed, we also paid attention to the meat quality of Huaxi cattle. Xia et al. identified 11 genes associated with meat quality traits, namely, *TMEM236* for fat color, *SORL1* and *TRDN* for meat color, *S100A10* and *AP2S1* for marble score, *KCTD16* and *LOC506594* for longissimus muscle area, as well as *DHX15* and *BRINP3* for shear force [9]. Fatty acid contents and components were also one of the important factors affecting the meat quality of Huaxi cattle. Zhu et al. found the *FASN* gene at 51.3 Mb on BTA19 could explain 6.49% and 10.04% of the genetic variances for C14:1 cis-9 and C14:0, respectively, and the *ELOVL5* gene was identified in 25.1 Mb regions on BTA23 explaining 1.5% of the genetic variance for C14:1 cis-9 [10].

Genomic selection technology has been widely used in Huaxi cattle breeding. Multiple strategies have been explored, including the additive dominance model [11], parallel Markov chain Monte Carlo [12], haplotype [13], elastic net [14], cosine kernel-based Kernel ridge regression (KCRR) [15], and stacked integrated learning framework [16], for the model optimization and method development to improve the accuracy of genomic selection. Selection signature analysis can reveal the underlying genetic mechanism of the formation of the new breed in genomic selective molecular breeding [17]. By the relative extended haplotype homozygosity (REHH) selection signatures scanning method using Illumina BovineSNP50 chip, *GHSR* related to body weight, *TG* associated with meat fat and marbling, and *CANCNA2D1* related to cattle flesh color, the dressing percentage and backfat thickness were screened [18].

Linkage disequilibrium (LD) is the non-random association of alleles at different loci. The LD patterns provide relevant information about past demographic events, which reflect the population history of natural and artificial selection [19]. Crossbreeding is a common strategy for the formation of modern livestock breeds. Compared to purebred, the LD level of crossbred populations is relatively lower [20,21]. However, previous studies have found that strongly artificial selection could result in small effective population sizes that facilitated the increase in LD within the population [22,23]. Huaxi cattle has experienced strong artificial breeding during the process of transverse fixup after the formation of the hybrid population. The population LD analysis can increase our understanding of the impact of recent strong artificial selection. Recently, selection signature analyses have greatly promoted the identification of genes associated with important economic and adaptive traits [24]. When selection occurs in the admixed populations, the selected alleles are expected to have a higher frequency and deviate from the genome-wide average after multi-generations of admixture, which reflects signatures of recent selection response [25]. Recently, various statistical methods have been developed to detect footprints of recent selection, including integrated haplotype score (iHS), composite likelihood ratio (CLR), cross-population extended haplotype homozygosity (XPEHH), and relative integrated EHH of a site between populations (Rsb). Application of these methods has greatly promoted the identification of candidate genes related to important economic traits in composite populations, such as Simbra crossbred [4], Vrindavani cattle [26], East African Shorthorn Zebu [27], and Swiss Fleckvieh cattle [25].

Nowadays, the intercrossing and trait fixation of Huaxi cattle has reached the fifth generation. A comprehensive assessment of its genetic diversity, population structure, and genomic selection signatures are necessary for uncovering genetic divergence among Huaxi cattle and other breeds. In this study, Illumina BovineHD SNP array was used to genotype nine populations, including Huaxi cattle, its ancestors, and similar populations. Then, the genetic diversity of nine populations was estimated by average minor allele frequency (*MAF*), observed heterozygosity (*Ho*), expected heterozygosity (*He*), and inbreeding coefficient (F_ROH_). Then, the population structure was elucidated by principal component analysis (PCA), neighbor-joining tree (NJ tree), population admixture, and (Nei) genetic distance analysis. Furthermore, the genomic selection signatures associated with important traits in the Huaxi cattle were determined based on the integrated haplotype score (iHS) and composite likelihood ratio (CLR) methods. Our study will help to evaluate the genetic background and plan the long-term breeding strategy of Huaxi cattle.

## 2. Materials and Methods

### 2.1. Sample Selection

To understand the genetic background and genomic selection signatures of Huaxi cattle, we collected 228 individuals from Huaxi cattle (HXC, *n* = 55), its maternal ancestors including Mongolian cattle (MGC, *n* = 20) and Sanhe cattle (SHC, *n* = 25), and its paternal ancestors including Charolais (CHL, *n* = 24), Fleevicht cattle (Sim_DEU, *n* = 25), American Simmental cattle (Sim_USA, *n* = 25), and Canadian Simmental cattle (Sim_CAN, *n* = 25), as well as two similar populations, Australian Simmental beef cattle (Sim_AUS, *n* = 21) and dual-purpose Montbeliard cattle from France (Sim_FR, *n* = 8). Sample details were presented in Table 1.

### 2.2. Genotyping and Quality Control

Genomic DNA was extracted from the blood samples of all cattle using the TIANamp Blood DNA Kit (Tiangen Biotech Company Ltd., Beijing, China). DNA was qualified when the DNA concentration was above 50 ng/uL and the OD260/280 ratio value ranged from 1.7 to 2.1. Genotyping was performed using the Illumina BovineHD SNP array that contained 777,962 SNPs. Single nucleotide polymorphisms (SNPs) were scanned using the iSCAN platform, and genotype calling was conducted using GenomeStudio software. Quality control of the raw data was performed using PLINK v1.9 software [28]. Individuals and SNP loci were discarded based on the following criteria: (1) SNPs with call rates less than 95%; (2) minor allele frequency (MAF) of SNPs less than 0.05; (3) significant deviation from Hardy–Weinberg equilibrium (*p* < 10^−6^); (4) individual with more than 10% missing genotypes; (5) closely related individuals were removed (PIHAT value > 0.25). In addition, only SNPs located on autosomal chromosomes were used for subsequent analysis [24].

### 2.3. Genetic Diversity, ROH Detection, and Linkage Disequilibrium Analysis

In order to assess the genetic diversity of nine diverse cattle populations, the average minor allele frequency (*MAF*), observed heterozygosity (*Ho*), and expected heterozygosity (*He*) were estimated with PLINK v1.9. In addition, we calculated the inbreeding coefficient based on ROH (F_ROH_) by the total length of individual ROH divided by the length of the autosomal genome with a sliding window approach using the default settings of “-homozyg” command [29,30]. To estimate and compare the genome-wide levels of linkage disequilibrium patterns among cattle groups, PopLDdecay v.3.40 software was used to calculate the square correlation coefficient (r^2^) between paired SNPs within 500 kb [31].

### 2.4. Population Structure and Phylogenetic Analysis

To ascertain the genetic relationship among Huaxi cattle and other breeds, we first pruned the marker set by excluding related SNP in a window of 50 SNP, sliding the window by 5 SNP, and r2 threshold of 0.2. A total of 63,510 SNPs were obtained after LD filtered to conduct the principal component analysis (PCA). Pairwise genome-wide identity-by-state (IBS) distances were estimated using PLINK v1.9, and the PCA graph was visualized through the ggplot2 R package. Then, we calculated the genetic distance matrix between pair-wise individuals. PHYLIP v3.69 was used to construct the neighbor-joining phylogenetic tree [32]. The phylogenetic tree was visualized with Figtree v1.3.1 [33]. To further evaluate the differences at the population level, the Nei’s genetic distance between Huaxi cattle and other populations was calculated by adegenet R package [34], and the NeighborNet network was constructed by SplitsTree v4.14.2 [35]. Population structure was examined for K = 2–5 using 11,349 SNPs after strict LD-based filter (r^2^ > 0.02) in STRUCTURE 2.3.4. Each process was performed with 10,000 burn-in cycles, followed by 10,000 replications under admixture and correlated allele frequency models [36].

### 2.5. Identification of Selection Signatures

To detect the genomic regions under selection in the breeding process of Huaxi cattle, two analysis methods including the integrated haplotype score (iHS) and composite likelihood ratio (CLR) test were applied for selection signature analysis. The iHS estimates the relative decay of extended haplotype homozygosity (EHH) of the ancestral and derived core allele [37,38]. The CLR test has a high detection effect on the variants near fixation, which quantifies significant deviations from the neutral site frequency spectrum (SFS) [39]. In this study, BEAGLE v5.2 (https://faculty.washington.edu/browning/beagle/beagle.html, accessed on 10 October 2021) with default settings was used to impute missing alleles and infer the haplotype phase for all individuals of Huaxi cattle [40]. The iHS was estimated using the default setting of selscan; although, the maximum gap was set to 800,000 [24,41]. We also applied the norm module of selscan to normalize the iHS score, and calculated the average |iHS| score in 100 kb non-overlapping windows across the autosomes. Regions at the top 1% with the highest average |iHS| score and SNP numbers greater than 10 were regarded as candidate regions of positive selection [42]. For the CLR test, we employed the software SweeD v3.2.1 (https://cme.h-its.org/exelixis/web/software/sweed/, accessed on 10 October 2021) to calculate the CLR values for sites every 20 kb across each chromosome of the genome [43]. To define candidate regions, we divided the genome into 100 kb non-overlapping windows. In each window, the maximum CLR value was used as the test statistic according to previous studies [44,45]. We used the 99th percentile of the distribution of CLR scores as the threshold for the detection of candidate regions.

### 2.6. Gene Annotation and Enrichment Analysis

Genes within the candidate regions were retrieved from the University of California Santa Cruz (UCSC) genome browser using the bovine UMD3.1 reference genome. To further investigate the potential biological function of selected genes in selected regions, we performed Gene Ontology (GO) function annotation and Kyoto Encyclopedia of Genes and Genomes (KEGG) pathway enrichment analysis by the Database for Annotation, Visualization and Integrated Discovery (DAVID) v6.8 [46]. Additionally, we further annotated candidate genes using the Cattle QTLdb (https://www.animalgenome.org/cgi-bin/QTLdb/BT/index, accessed on 10 October 2021) to detect whether the selected genes have been reported to be associated with important functional traits of cattle.

## 3. Results

### 3.1. Genetic Diversity

After quality control, all samples showed high genotyping call rates (call rate > 95%) and low inter-individual relatedness (PIHAT value < 0.25). Finally, a total of 592,920 autosomal SNPs of 228 individuals were obtained for subsequent analysis. We calculated the average minor allele frequency (*MAF*), average observed heterozygosity (*Ho*), average expected heterozygosity (*He*), and inbreeding coefficient based on ROH (F_ROH_) to assess polymorphism of nine cattle populations. As shown in Table 1, we observed that the average MAF of nine populations ranged from 0.235 to 0.290. The *MAF* of the Huaxi cattle population was 0.256, which was between Montbeliard cattle and Canadian Simmental cattle. Among all groups in this study, the *Ho* of Huaxi cattle was 0.375, which was between Mongolian cattle and Sanhe cattle. The *He* of Huaxi cattle was 0.34, which was between Canadian Simmental cattle and Montbeliard cattle. Overall, the value of *He* was considerably lower than the value of *Ho* in each population, except for Mongolian Cattle, a native breed of China. The F_ROH_ value of Huaxi cattle was 0.053, which was lower than that of the three commercial beef Simmental cattle groups from American (0.15), Canadian (0.094), and Australian (0.071), and approximate to that of Mongolian cattle (0.049), Sanhe cattle (0.052), and Fleevicht cattle (0.053). Moreover, Charolais has the maximum values of each parameter of genetic diversity and the minimum value of inbreeding coefficient when compared with other cattle populations, while American Simmental cattle were the opposite.

### 3.2. Linkage Disequilibrium

**Figure 2:**
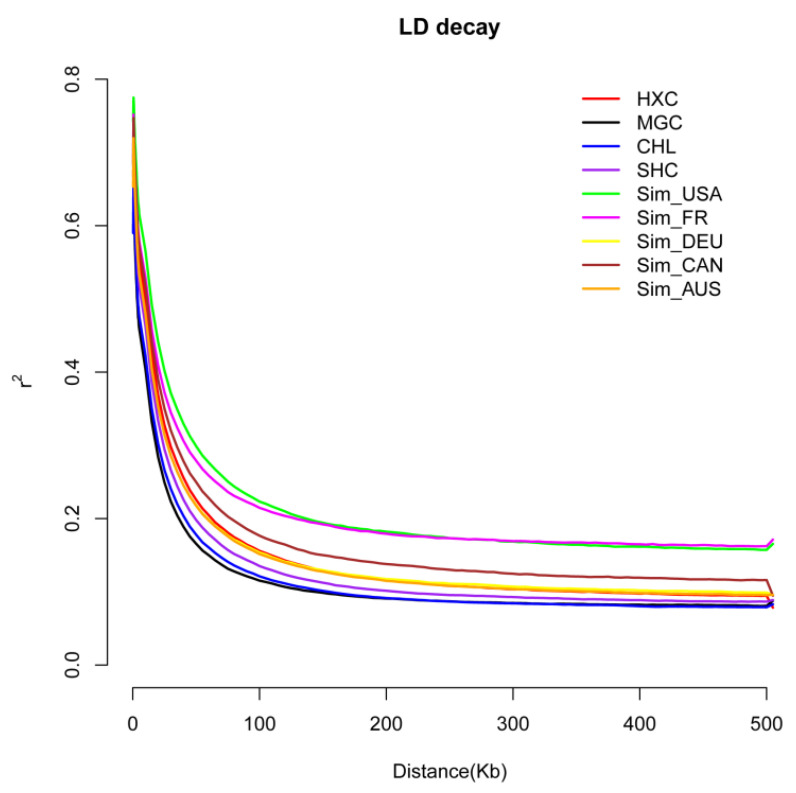
The linkage disequilibrium (LD) decay analysis of the nine cattle populations.

The effect of artificial selection could be reflected in the genome linkage disequilibrium (LD) levels in each population [23]. To measure the selection intensity of Huaxi cattle in the breeding process, linkage disequilibrium analyses were conducted on the nine populations. As shown in Figure 2, the LD attenuation rate of Mongolian cattle was the fastest, followed by Charolais cattle and Sanhe cattle. The attenuation rate of LD of Simmental cattle bred from six different countries was relatively slow, among which the attenuation rate of American Simmental cattle and Montbeliard cattle was the slowest, while the decline rate of linkage disequilibrium of Huaxi cattle was between Fleevicht cattle and Canadian Simmental cattle. Studies have shown that artificial selection promotes the increase in LD levels within the population [47,48]. Compared with two local breeds, such as Charolais and Mongolian cattle, the LD decay of Huaxi cattle was relatively slower after four generations of selective breeding. While Huaxi cattle showed a faster LD decay relative to the bred Simmental cattle populations such as Montbeliard cattle, Canadian Simmental cattle, and American Simmental cattle that have undergone a long period of artificial selection and mating.

### 3.3. Population Structure, Admixture, and Phylogenetic Analysis

**Figure 3:**
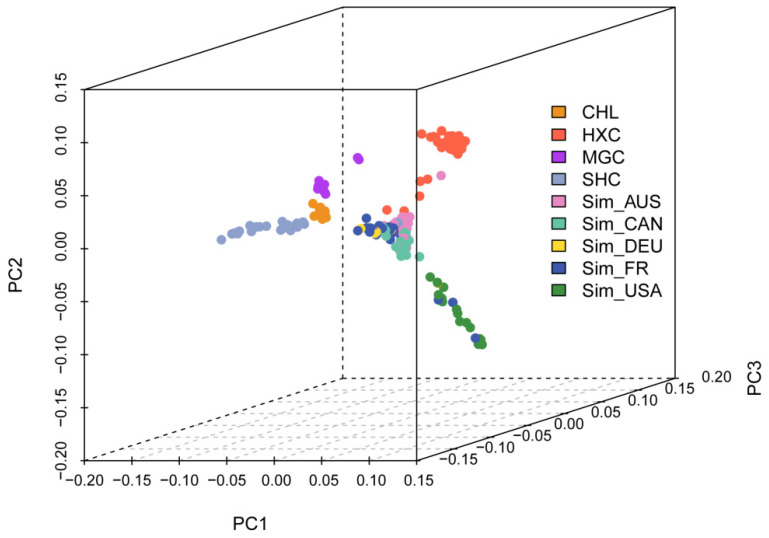
The principal component analysis (PCA) result of 228 individuals from nine cattle populations.

To investigate the cluster patterns among the animals analyzed, principal component analysis (PCA) was conducted (Figure 3). In the first dimension, Huaxi cattle and the five Simmental cattle populations obviously separated from non-Simmental lineage cattle (Mongolian, Charolais, and Sanhe cattle). The second dimension distinguished Huaxi cattle from the five Simmental herds, and the results indicated that the individuals of the Huaxi cattle, Mongolian cattle, Sanhe cattle, and Charolais cattle were gathered separately.

**Figure 4:**
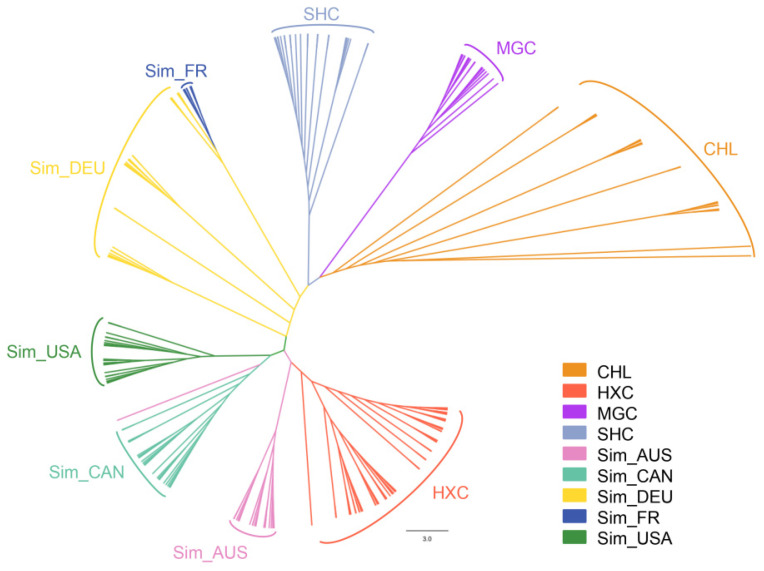
Neighbor-joining phylogenetic tree of 228 individuals from nine cattle populations.

Then, a neighbor-joining tree was constructed using the pairwise individual genetic distance matrix. Consistent with the PCA result, phylogenetic analysis showed that the individuals from the Huaxi cattle group were roughly clustered together and formed an independent branch, presenting high genetic consistency (Figure 4). Moreover, Huaxi cattle had the closest genetic relationship with beef Simmental cattle from Australian, American, and Canadian, followed by dual-purpose Simmental cattle (Fleevicht cattle and Montbeliard cattle), and far from Sanhe cattle, Mongolian cattle, and Charolais cattle.

**Figure 5:**
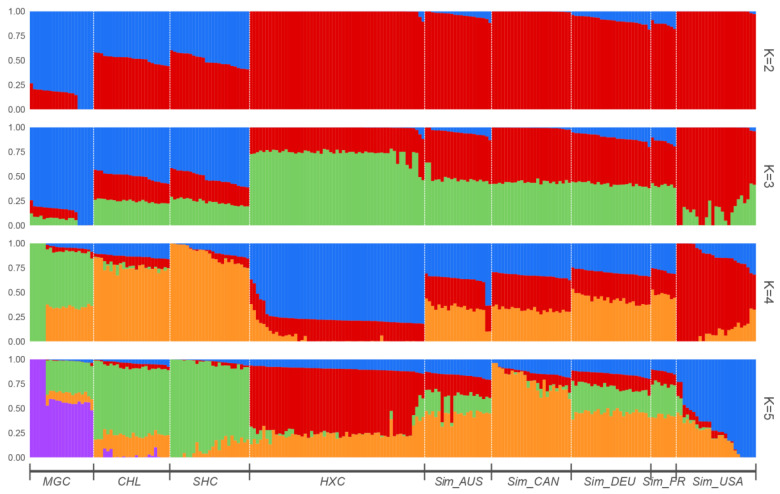
Structure analysis based on LD filtered SNPs for nine cattle populations when K = 2 to 5.

Next, to determine admixture degree in the nine cattle populations, STRUCTURE v2.3.4 software was used to perform the population admixture analysis. The hypothetical ancestral groups ranged from K = 2 to 5 (Figure 5). At k = 2, the clustering pattern implied that Huaxi cattle had high similarity with the five Simmental cattle populations and remarkable division from Mongolian Cattle, Sanhe cattle, and Charolais cattle. At K = 3, American Simmental cattle seemed to display different proportions of the genetic components compared to Huaxi cattle and other Simmental cattle populations. At K = 4, we observed that Australian Simmental cattle, Canadian Simmental cattle, Fleevicht cattle, and Montbeliard cattle sharing a higher degree of genetic similarity, while Huaxi cattle displayed different admixture component proportions among all populations, indicating that Huaxi cattle was distinguished from the other breeds and had its own unique lineage composition.

### 3.4. Population Genetic Distance Measure

**Figure 6:**
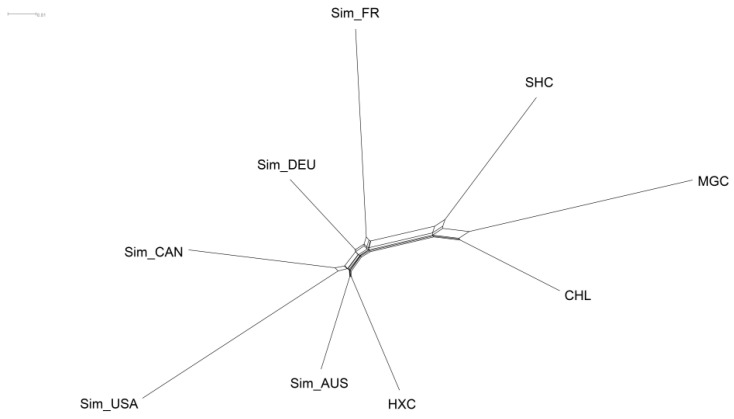
NeighborNet graph constructed based on Nei’s genetic distance between nine cattle populations.

To explore the genetic variation at the population level, we further evaluated Nei’s genetic distance among the nine populations. The results showed that the Nei’s distance between the nine populations ranged from 0.0775 to 0.2103 (Table S1). Among them, Huaxi cattle had the smallest genetic distance with Australian Simmental cattle (0.0775) and the largest genetic distance with Mongolian cattle (0.1737). The NeighborNet graph was then constructed based on Nei’s genetic distance values (Figure 6). We observed that Huaxi cattle, Australian Simmental cattle, Canadian Simmental cattle, and American Simmental cattle were in the same sub-cluster, as well as Fleevicht and Montbeliard cattle were in another sub-cluster. In accordance with the results of NJ tree and PCA, Mongolian cattle, Sanhe cattle, and Charolais cattle have a relatively far genetic distance from Huaxi cattle.

### 3.5. Identification of Selection Signatures

**Figure 7:**
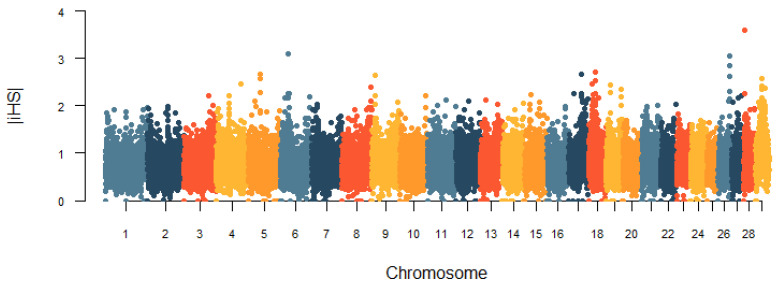
Genome-wide distribution of the integrated haplotype score (iHS) values in Huaxi cattle.

**Figure 8:**
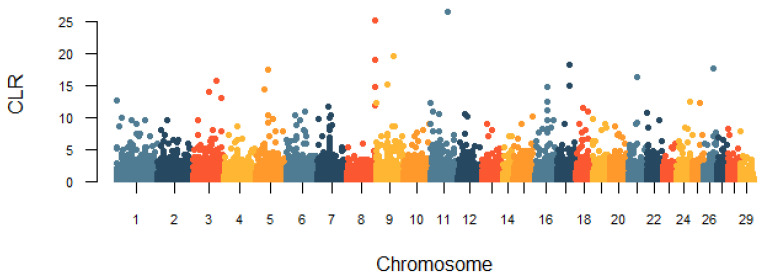
Genome-wide distribution of the composite likelihood ratio (CLR) values in Huaxi cattle.

In this study, the genomic regions under the recent selection of the Huaxi cattle were determined by two methods, iHS and CLR. The genome-wide distribution of |iHS| values for 100 k non-overlapping windows on autosomes is depicted in Figure 7. In total, there were 248 candidate regions under the threshold of the top 1% that were identified, and we obtained 143 genes under selection in the iHS test (Table S2). Based on the cattle QTLdb database, we found that 14 genes were associated with important economic traits of cattle, including growth and development, carcass and meat quality, and milk quality (Table 2). The CLR statistic for 100 k non-overlapping windows in the genome is shown in Figure 8. We identified 247 positive selection regions with extreme CLR values using the top 1% criteria for the CLR test. The 68.6–68.7 Mb region of BTA11 had the highest CLR value (CLR = 26.57). Through gene retrieval, 199 potentially selected candidate genes were obtained in the CLR test (Table S3). Among them, we also observed some candidate genes related to reproduction, growth and development, carcass and meat quality, and immunity (Table 2). Notably, nine genes (*KCNK1*, *PDLIM5*, *CPXM2*, *CAPN14*, *MIR2285D*, *MYOF*, *PKDCC*, *FOXN3*, and *EHD3*) in fifteen overlapping genomic regions covered 1.5 Mb were jointly detected by both methods.

We further performed the GO function annotation and KEGG pathway analysis on the selected genes. As shown in Table S4, a total of 20 GO terms with *p*-value < 0.05 were observed. Among them, genes were significantly enriched in metabolism-related and immune-related biological functions, such as interleukin-1-mediated signaling pathway (GO:0070498, *p* = 0.018), B-1a B cell differentiation (GO:0002337, *p* = 0.035), negative regulation of interleukin-6 production (GO:0032715, *p* = 0.043), pantothenate metabolic process (GO:0015939, *p* = 0.035), proteolysis (GO:0006508, *p* = 0.036). In addition, we obtained five significant enriched pathways, including ubiquitin mediated proteolysis (bta04120, *p* = 0.01), PI3K-Akt signaling pathway (bta04151, *p* = 0.019), pantothenate and CoA biosynthesis (bta00770, *p* = 0.039), nicotine addiction (bta05033, *p* = 0.037), and pathways in cancer (bta05200, *p* = 0.047), which were related to immune, metabolism, and signal transduction.

## 4. Discussion

Huaxi cattle is a new specialized beef cattle breed that has been bred by Chinese breeders for 43 years. Due to its fast growth rate, rough feeding resistance, wide adaptability, and good economic effect, Huaxi cattle is now widely distributed in many provinces of China, such as Inner Mongolia, Jilin, Henan, Hubei, Yunnan, and Xinjiang, greatly promoting the increase in beef cattle industry and farmers’ economic income in China. In this study, to better understand the characteristics of Huaxi cattle, we comprehensively evaluated the genetic diversity, population structure, admixture, and phylogenetic relationship by comparing Huaxi cattle with its ancestors and similar populations.

Genetic diversity is the basis for species evolution to adapt to the environment and an important reference index to evaluate the status of germplasm resources [49]. Our analyses revealed the MAF, expected heterozygosity, observed heterozygosity, and inbreeding coefficient of Huaxi cattle were 0.2557, 0.3754, 0.3402, and 0.0535, respectively, which were at the medium level of polymorphism information content among the nine cattle populations in this study. It has been reported that the heterosis produced by hybridization of parental inbred lines conferred the offspring higher genetic diversity [50,51]. We found that the observed heterozygosity of Huaxi cattle exceeded the expected heterozygosity under the Hardy–Weinberg equilibrium. Hence, crossbreeding is still an important method to expand the genetic variation of modern cattle breeds [4]. High-intensity artificial selection might promote genetic progress, but it could lead to an increase in the population inbreeding level [52]. Huaxi cattle have undergone generations of artificial selection in the breeding process in recent years. So far, the inbreeding level of Huaxi cattle is relatively low compared to the three commercial beef Simmental cattle populations in America, Canada, and Australia. Additionally, LD decay patterns of different breeds reflected the corresponding unique breeding selection history and population structure, and artificial selection can promote the increase in LD in the population [23,53,54]. We observed that Huaxi cattle showed a faster LD decay rate relative to Montbeliard cattle, Canadian Simmental cattle, and American Simmental cattle, which indicated that the degree of selection intensity of Huaxi cattle was lower than that of commercial Simmental cattle.

PCA and NJ tree analyses were further conducted to reveal the genetic relationship and genetic differentiation among Huaxi cattle and other breeds. The results showed that the individuals from Huaxi cattle gathered clearly and obviously separated from other cattle breeds. STRUCTURE analysis also demonstrated that Huaxi cattle has formed various genetic backgrounds compared to other breeds. Furthermore, we calculated the genetic distance among different cattle herds at the population level. We found that the genetic relationship between Huaxi cattle and beef Simmental cattle was slightly close, and far from Mongolian cattle, Sanhe cattle, and Charolais cattle, which was consistent with the results of the phylogenetic tree. Overall, after the initial population formation and subsequent artificial selection in the breeding, the Huaxi cattle population has been genetically stable over time and developed into a unique breed.

Natural selection or intensively artificial selection may have left selection footprint on the genomes of individuals within the population [55]. Identifying selection signatures provide a new perspective for understanding the genetic mechanisms controlling specific phenotypes under selection, and a better guide in animal breeding [56,57]. In this study, iHS and CLR, two sensitive detection methods for recent selection signatures, were used to identify candidate genomic regions with signatures under positive selection. Due to the diversity of the target genomic genetic variations [58], iHS test had a high detection effect on recent selective sweeps with intermediate frequency variation [59], while CLR was more powerful in detecting high frequency or approaching fixed selective sweeps [39,40]. We identified 247 and 248 candidate gene regions, respectively, of which 1.5 Mb selection area overlapped in the two selective signature detection methods.

An important goal of this study was to identify candidate genes involved in the domestication and artificial selection of Huaxi cattle. Through gene retrieval, we found that some genes were related to important functional traits in the potential selected regions. For example, *TBC1D5*, *CDK6*, *RXRA*, *LCORL*, and *CAPN2* genes were involved in growth and development [6,24,60,61,62,63,64,65,66,67]; and *ZNF280B*, *CA10*, *LAP3*, *POLB*, *HELB*, *IRAK3*, and *ANO5* genes were associated with carcass and beef quality [8,67,68,69]. By enrichment analysis, we also discovered that the potential selected genes in Huaxi cattle were significantly enriched in metabolism functions, such as pantothenate metabolic process, proteolysis, PI3K-Akt signaling pathway, and pantothenate and CoA biosynthesis, which suggest that genes related to growth and development and meat quality are most likely under selection in Huaxi cattle. Indeed, growth rate, and beef quantity and quality have always been the main focus in the breeding process of Huaxi cattle. Strong resistance to disease was the basis for the extensive adaptability of Huaxi cattle in vast areas of China. Moreover, we found that the identified selected genes were significantly enriched in immune-related functions, including interleukin-1-mediated signaling pathway, B-1a B cell differentiation, and negative regulation of interleukin-6 production. Of which, *KDR*, a receptor for vascular endothelial growth factor, has been reported to be associated with susceptibility to bovine respiratory disease [70]. In addition, nine shared genes were detected by both methods. *CAPN14* and *EHD3* are associated with calcium ion binding [71,72], and calcium ions are minerals essential for bone growth. *CPXM2* regulates the early differentiation of connective tissues and has been recognized to be a gene associated with fetal growth restriction [73,74]. *MYOF* is a member of the Ferlin family involved in the recycling of insulin-like growth factor (IGF) receptor, which plays major roles in controlling somatic growth and participate in skeletal muscle growth and differentiation [75]. It has been found to be selected in the Japanese population [76]. *PDLIM5*, a cytoskeleton-related protein that tethers protein kinases to the Z-disk in striated muscles, plays a key role in the proliferation and differentiation of the skeletal muscle, nervous, myocardium, and tumor [77]. The *PKDCC* gene is a putative protein kinase that has been reported to be involved in embryonic development and plays an essential role in bone development of the human and mouse [78,79]. *KCNK1* belongs to the family of background K channel [80]. *FOXN3* was identified as a selected candidate gene, which has been reported to be involved in cell proliferation, apoptosis, and pathogenesis in cancer, and functions as a tumor suppressor [81]. According to their functions, we speculate that these potentially selected genes may have a role in the production traits and environmental adaptability of Huaxi cattle.

## 5. Conclusions

In this study, we confirmed that Huaxi cattle has formed its own unique genetic structure feature, and we identified numerous candidate genes that have potential effects on important phenotypic traits such as growth and development, meat quality, and immune response. Our findings will conduce to a comprehensive understanding of the unique genetic features and phylogenetic relationships in Huaxi cattle, and provide a new perspective on the genetic mechanisms controlling specific phenotypes under selection.

## Figures and Tables

**Table 1:** The minor allele frequency, observed and expected heterozygosities and inbreeding coefficient based on ROH in nine cattle populations.

Population	Abbreviation	Number	MAF ^1^	Ho ^2^	He ^3^	F_ROH_ ^4^
Mongolian cattle	MGC	20	0.289	0.366	0.377	0.049
Sanhe cattle	SHC	25	0.280	0.378	0.366	0.052
Charolais	CHL	24	0.290	0.388	0.377	0.034
Australian Simmental cattle	Sim_AUS	21	0.267	0.365	0.352	0.071
Canadian Simmental cattle	Sim_CAN	25	0.252	0.357	0.334	0.094
American Simmental cattle	Sim_USA	21	0.235	0.334	0.313	0.150
Fleevicht cattle	Sim_DEU	25	0.273	0.384	0.358	0.053
Montbeliard cattle	Sim_FR	8	0.261	0.387	0.342	0.046
Huaxi cattle	HXC	55	0.256	0.375	0.340	0.053

^1^ Minor Allele Frequency. ^2^ Observed Heterozygosity. ^3^ Expected Heterozygosity. ^4^ ROH based Inbreeding Coefficient.

**Table 2:** Potential selected genes associated with important economic traits in Huaxi cattle ^1^.

Trait Class	Trait	Gene Detected by iHS	Gene Detected by CLR
Growth and development	Average daily feed intake		LCORL
	Body length	CDK6	CAPN2
	Body weight	RXRA, TBC1D5	CAPN2
Carcass and meat quality	Carcass weight	ZNF280B	CA10, LCORL
	Bone weight		LAP3, LCORL
	Bone quality	POLB	
	Marbling score		HELB, IRAK3
	Longissimus muscle area		LCORL
	Fat thickness at the 12th rib	RXRA	LCORL
	Monounsaturated fatty acid content	RXRA	
	Meat texture	ANO5	
Reproduction	Conception rate		DZIP3
	Daughter pregnancy rate		AMN1, COQ9, KCNMB2, CACNA2D3
	Early embryonic survival		SLC18A2
Milk	Milk yield	NCKAP1L	ABCA7, DNAJC21, IL20RA
	Milk fat yield	TBX5, CNOT1, NDRG4, NCKAP1L, CNOT1	AMN1, NELL2, PCED1B
	Milk protein yield	GRIN3A, NDRG4, VPS35	DZIP3, IL12RB2, LAP3, MED28, SPSB1, CACNA2D3
	Milking speed		SLC18A2
	Milk C14 index	ANO5	
	Milk-conjugated linoleic acid content	ASIC2	
	Milk cholesterol content	RBM19	
Health	Bovine respiratory disease susceptibility		KDR

^1^ This table is based on the information retrieved in the Cattle QTLdb database (accessed on 10 October 2021).

## Data Availability

The data presented in this study are available on request from the corresponding author. The data are not publicly available to preserve the privacy of the data.

## Supplementary Materials

The following are available online at https://www.mdpi.com/article/10.3390/ani11123469/s1, Table S1: Pair-wise Nei’s distance among nine cattle populations, Table S2: A summary of genes from iHS in Huaxi cattle, Table S3: A summary of genes from CLR in Huaxi cattle, Table S4: GO and KEGG enrichment analysis of Huaxi cattle candidate genes by iHS and CLR methods.
